# A novel intrarenal multimodal 2D/3D registration algorithm and preliminary phantom study

**DOI:** 10.1002/acm2.14084

**Published:** 2023-07-10

**Authors:** Zuoming Fu, Ziyi Jin, Chongan Zhang, Peng Wang, Hong Zhang, Xuesong Ye

**Affiliations:** ^1^ Biosensor National Special Laboratory, College of Biomedical Engineering and Instrument Science Zhejiang University Hangzhou China; ^2^ Hangzhou Xianao Technology Co., Ltd Hangzhou China

**Keywords:** multimodal image registration, multi‐view constraint, retrograde intrarenal surgery, structural feature extraction, style transfer

## Abstract

Retrograde intrarenal surgery (RIRS) is a widely utilized diagnostic and therapeutic tool for multiple upper urinary tract pathologies. The image‐guided navigation system can assist the surgeon to perform precise surgery by providing the relative position between the lesion and the instrument after the intraoperative image is registered with the preoperative model. However, due to the structural complexity and diversity of multi‐branched organs such as kidneys, bronchi, etc., the consistency of the intensity distribution of virtual and real images will be challenged, which makes the classical pure intensity registration method prone to bias and random results in a wide search domain. In this paper, we propose a structural feature similarity‐based method combined with a semantic style transfer network, which significantly improves the registration accuracy when the initial state deviation is obvious. Furthermore, multi‐view constraints are introduced to compensate for the collapse of spatial depth information and improve the robustness of the algorithm. Experimental studies were conducted on two models generated from patient data to evaluate the performance of the method and competing algorithms. The proposed method obtains mean target error (mTRE) of 0.971 ± 0.585 mm and 1.266 ± 0.416 mm respectively, with better accuracy and robustness overall. Experimental results demonstrate that the proposed method has the potential to be applied to RIRS and extended to other organs with similar structures.

## 1 INTRODUCTION

Nephrolithiasis is one of the most common urological diseases in recent years. Its prevalence is increasing over the last two decades and is highly recurrent, with recurrence risks as high as 50% within 10 years. As a diagnostic and therapeutic tool in the management of upper urinary tract pathologies including nephrolithiasis, retrograde intrarenal surgery (RIRS) has been widely utilized.^1^, ^2^, ^3^

In RIRS, the surgeon usually needs to use the ureteroscope to search the calyces successively until the target is located.^4^ Novice surgeons are often disoriented and less effective due to the tortuous anatomy of the renal cavity and poor visibility due to microhematuria. Surgeons generally use fluoroscopic guidance to regain their spatial orientation, but this would expose the patient to the risk of excessive radiation. In addition, intraoperative two‐dimensional (2D) images only provide a limited viewing angle, which has disadvantages such as lack of depth information, occlusion, and dependence on experience. The presence of the above factors will inevitably lead to an increase in operative time and tissue injury, which is a crucial risk factor for some severe complications, such as septic shock, cardiovascular events, and blood loss.^5^, ^6^, ^7^ The development of a surgical navigation system offers hope for a solution to the abovementioned problems.

The surgical navigation system is based on the preoperative medical image (CT, MRI, etc.), reconstructs the three‐dimensional (3D) model, and combines with the real‐time tracking system to guide the operation of doctors. As a crucial part of the navigation system for RIRS, endoscopic 2D/3D registration realizes the coordinate system alignment between the 3D model and the intraoperative camera.

The problem of 2D/3D registration has been widely studied and a large number of methods were proposed.^8^, ^9^, ^10^, ^11^, ^12^ They were mainly classified into three categories: landmark‐based registration, computer vision and deep learning‐based registration, and intensity‐based registration.

Landmark‐based registration is realized by establishing and aligning correspondence (landmarks/features) between objects. The principle is simple and efficient, but its effectiveness depends on the matching accuracy of the correspondence. For the acquisition of landmarks, the previous work mainly relied on the discrimination and extraction of human eyes. Recently, automatic landmark detection methods based on deep learning networks have also emerged. In general, extracting feature landmarks from complex anatomical shapes is an extremely challenging problem, especially for flexible internal organs of the human body. This approach mainly focused on rigid parts such as brains, bones, and teeth.

With the rapid development of computer vision and deep learning, the above technologies have also been applied to registration. Early research mainly uses traditional depth reconstruction algorithms (SFS, SFM, SLAM, etc.) to construct surface point clouds and convert 2D/3D problems into 3D/3D registration.^13^, ^14^ In recent years, learning‐based depth prediction networks^15^ and end‐to‐end registration networks^16^ have emerged for solving the registration problem. However, Unberath et al. believed that deep learning is still in the exploration stage, and the generalization ability of the network model on different patients remains to be verified. Therefore, a hybrid registration guided by traditional methods and combined with deep learning may be a good choice.^17^ Koo et al.^18^ and Labrunie et al.^19^ successively used the segmentation network for contour feature extraction and then used the RANSAC‐PNP algorithm for direct 2D/3D registration.

The intensity‐based registration is one of the important contents of 2D/3D endoscopic registration. Usually, intensity‐based registration usually requires a robust similarity function to accurately characterize the intensity difference to guide image matching. Helferty et al. proposed a normalized mutual information (NMI)‐based global similarity measurement to match the bronchoscopic image and virtual image.^20^ The work^21^, ^22^, ^23^ successively explored and presented intensity‐based constraints to perform registration in the bronchi, digestive tract, etc. More recently, research work^24^, ^25^ proposed an HWD‐driven similarity measure and a multiscale structural similarity measure for bronchoscopic registration, respectively. However, the performance of this method depends on the intensity consistency between real images and virtual images and is easily affected by differences in multimodal data.

Although 2D/3D registration has been extensively studied, it mainly focuses on the brain, orthopedics, bronchus, ear, throat, etc. For registration in the intrarenal cavity, there are still the following two problems: (1) Methods suitable for intrarenal cavity registration, including the determination of structural features, the establishment of similarity measures, or methods based on deep learning remain to be studied; (2) the complexity and diversity of intrarenal structures, the lack of significant and robust landmarks, and the complicated intraoperative environment bring challenges to registration. For example, in complex intrarenal structures, it is difficult to maintain intensity consistency between virtual and real images. Classical intensity‐based registration methods tend to show biased results and get trapped in local minima.

Therefore, in this article, we define a projected structural contour feature and propose a robust and high‐precision 2D/3D registration framework for addressing the initial registration in RIRS surgery. The framework effectively uses the structural features of renal anatomy and endoscopic images to automatically extract 3D feature points and corresponding 2D distinctive regions for matching. In addition, an image translation network is introduced to mitigate the impact of multimodal data, and multi‐view constraints are added to compensate for the collapse of spatial depth information to improve robustness and accuracy. Moreover, an experimental study was performed on two models with significant shape differences to evaluate the performance of the method and compare it with other competing algorithms.

## 2 MATERIALS AND METHODS

### 2.1 Algorithm overview

**FIGURE 1:**
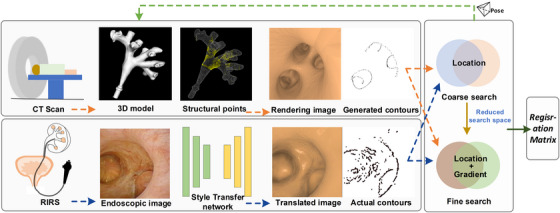
The working pipeline of the proposed registration framework

The developed algorithm includes preoperative 3D structural feature extraction, 2D image salient region extraction, iterative projection of structural features, and optimal transformation search based on similarity measure. The 3D structure features are sparse representations of salient regions in the CT volume, and its 2D projections have a strong gradient on the image. The 3D feature projections and 2D distinctive points are respectively defined as generated contours and actual contours. Intuitively, the registration is achieved by minimizing visual misalignment between the generated contours and actual contours. Generated contours are the projection from an iterated estimated pose. A similarity cost function is established and a coarse‐to‐fine search strategy was employed to estimate the optimal transform. Furthermore, a style transfer network is integrated to mitigate the impact of differences between multimodal data. The collapse of spatial depth information makes it difficult to obtain precise 2D/3D registration through 2D‐2D contour matching. Thus, a multi‐view constraint is added to improve the accuracy and robustness. To sum up, the main modules and processing pipeline are shown in Figure 1.

### 2.2 Assumptions and initiation

**FIGURE 2:**
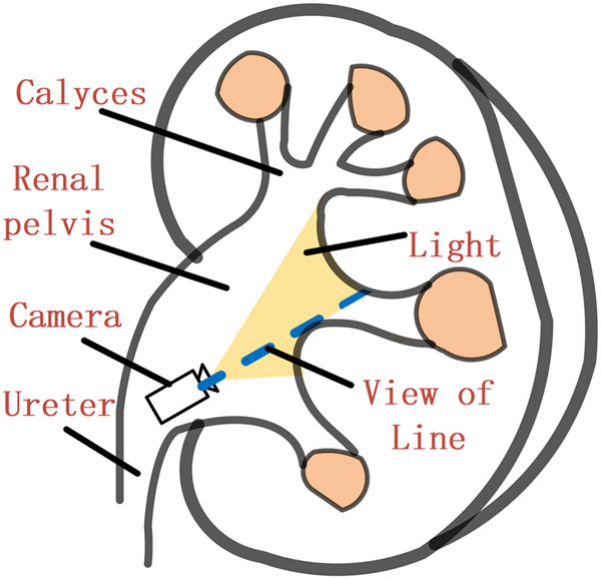
A typical working scenario in surgery. Light occlusion from an endoscopic perspective.

**FIGURE 3:**
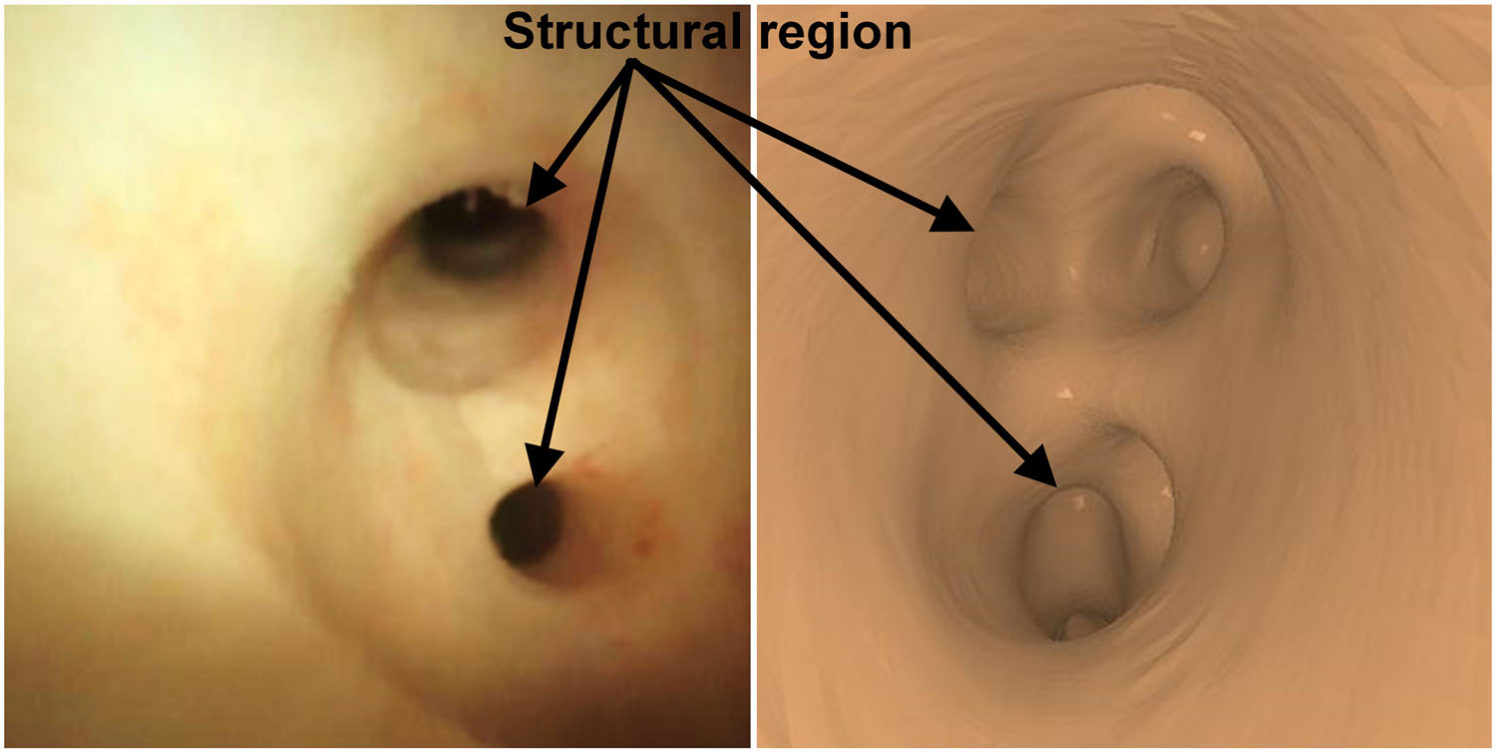
The structural region in the real and the virtual rendered image.

A key assumption of our algorithm is that a common set of feature points can be robustly extracted from the 3D volume and 2D images. Feature points in 2D images are generated due to the interaction of anatomical structure, illumination, and line of sight. A typical renal collection system and the state of the endoscope observing the calyces is shown in Figure 2. For 3D surface points with gradients perpendicular to the line of sight, their projected contours have significant intensity differences compared to their surroundings due to light occlusion effects, especially the edge of minor calyces and the joints of the minor and major calyces. This phenomenon can be observed in both real and virtual rendered images, as shown in Figure 3.

The task of 2D/3D registration is to find a high‐precision transformation $T\in R^{4\times 4}$ between the volume and the camera space. Searching in an entire volume space would take a lot of effort. Obtaining an initial pose estimation $T_{0}\in R^{4\times 4}$ would reduce search space. It does not necessarily ensure an accurate matrix. Therefore, in our work, the landmark‐based registration is performed manually to get the *T*_0_.

### 2.3 Salient feature points extraction

**FIGURE 4:**
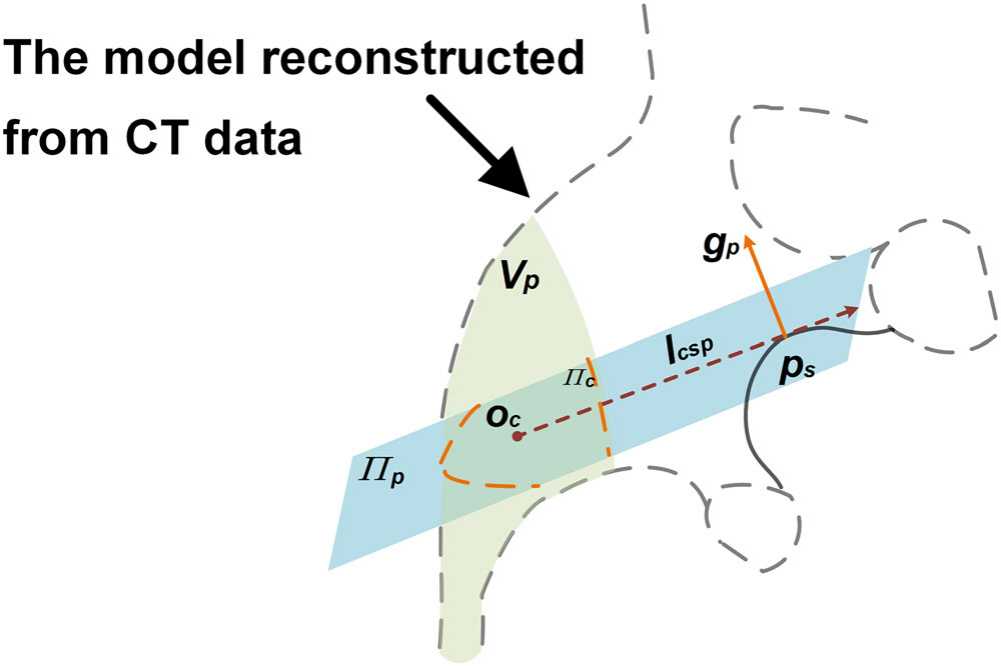
Schematic diagram of salient feature point extraction in a three‐dimensional (3D) model. The *p_s_* is a feature point in this figure.

**FIGURE 5:**
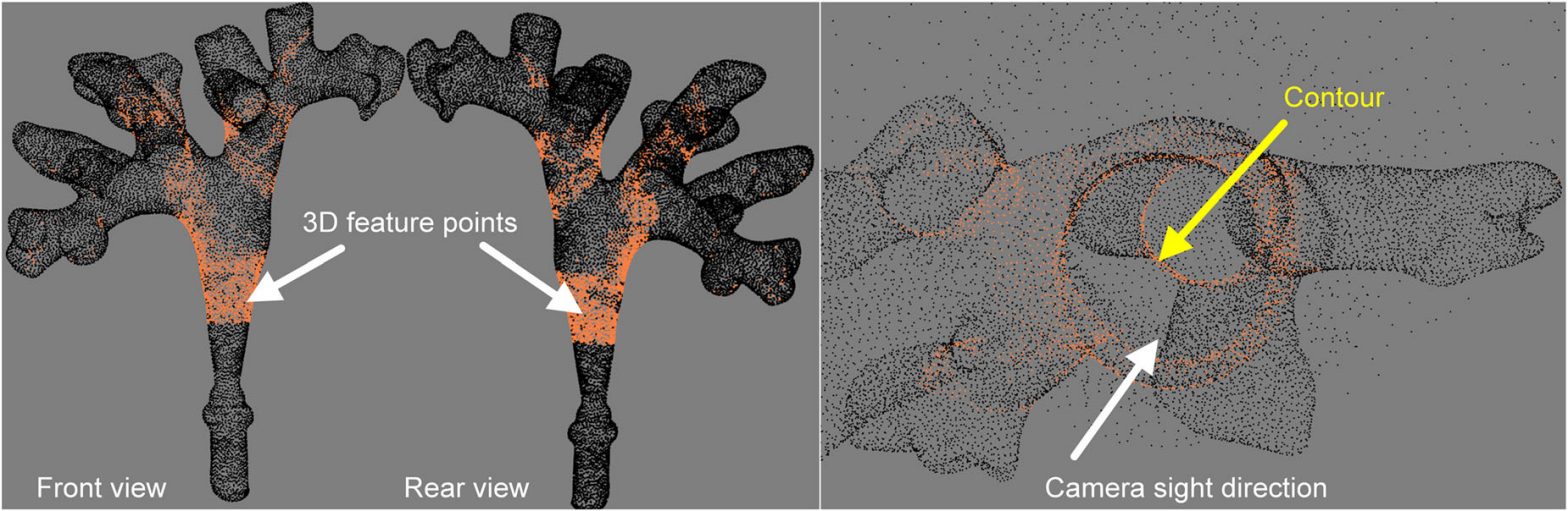
Structural feature points in front view, rear view, and camera view.

As shown in Figure 4, to observe more obvious structural features, a search space $V_{p}$ is defined in the renal pelvis to achieve initial registration. For each camera pose $T_{c}\in V_{p}$, a salient point set $P_{T_{c}}$ related to $T_{c}$ was selected from the CT model surface. $P_{T_{c}}$ has the following characteristic: (1) at the camera pose $T_{c}$, through the camera projection model, a 2D contour can be generated on the image; (2) satisfies perpendicularity and visibility conditions. Further, the salient points $F_{V_{p}}$ of the entire model consists of a series of $P_{T_{c}}$, $F_{V_{p}}={\left\{P_{T_{c}}\right\}}_{T_{c}\in V_{p}}\;$. $F_{V_{p}}$ are usually distributed continuously in the prominences of the kidney, such as the junction of the pelvis and calyces, and the junction of the major and minor calyces, as shown in Figure 5.

(1) Intersection: The salient structure points $p_{s}$ have the characteristics that the normal $\vec{g_{p}}$ is perpendicular to the viewing vector $\vec{w}$. In this case, the optical center $o_{c}$ is constrained to be on the tangent plane π_p_, fulfilling the point‐on‐plane condition. An intersection test is performed between the tangent plane π_p_ and the renal pelvis space $V_{p}$ to determine the common spatial region π_c_. If the region π_c_ exists, then the collision test would be performed. An intuitive explanation is shown in Figure 4.

(2) Collision: The prerequisite for observing $p_{s}$ from $o_{c}\;$successfully is that there are no obstacles on the line segment $l_{cp}$ formed by $o_{c}$ and $p_{s}$. The purpose of this test is to determine whether there is point $o_{c}$ on the plane π_p_. The triangles of the volume are considered as obstacles. Specifically, we create a number of seed points in plane π_p_ with equal intervals. For each seed point $o_{cs}$, we calculate the line segment $l_{csp}$ with $p_{s}$ and execute the collision detection between rays and triangles. When no collision occurs, the $o_{cs}$ is marked as a valid point. When the number of valid points is greater than the threshold $N_{s}$, $p_{s}$ was determined as a significant feature point.

### 2.4 D. Salient point selection with the iterated pose

During the registration process, the generated contours are continuously updated according to the iterative camera pose $T_{last}$. The perpendicularity and visibility test were performed to select the set of salient points $P_{T_{last}}$ from $F_{V_{p}}$. And then, the $P_{T_{last}}$ is projected to obtain the up‐to‐date generated contours.

(1) Perpendicularity: It is worth noting that this requirement is the first necessary condition. For each point in the feature points subset *F*, we select points that satisfy the following angle constraint

(1) $$\theta_{i}=\arccos\left(\left|\frac{\left|\vec{g_{i}}\;\cdot \;\vec{w_{i}}\right|}{\left|\vec{g_{i}}\right|\cdot \left|\vec{w_{i}}\right|}\right|\right)\;\geq \theta_{T}$$

where $\theta_{i}$ is the angle between the normal of the salient point and the viewing ray, $\vec{g_{i}}$ is the normal of point and $\vec{w_{i}}$ is the direction of viewing ray. The perpendicular threshold $\theta_{T}$ controls the contour thickness and is empirically set close to 90◦.

(2) Visibility: This test is used to check whether a salient feature point can be observed on the 2D projection image. A collision detection algorithm is performed similarly in the last section. We detect whether there are obstacles on the path between the salient point and the viewing position.

### 2.5 E. Semantic GAN‐based translation

In this paper, the images $I^{endo}$ and $I^{ct}$ used for similarity matching were derived respectively from intraoperative endoscopic images and virtual rendering images. Referring to the standard virtual endoscopy techniques, $I^{ct}$ was generated using the surface rendering method under the OpenGL framework. Moreover, there are two aspects that need to be matched with the real endoscope for registration application: (1) camera model parameters related to the image projection, including focal length, the field of view, radial distortion, and principal point. (2) Lighting model for scene rendering, including the light source and scene lighting. The Phong lighting model was used to simulate complicated scenarios. The lighting parameters used were selected by visual inspection to match the real image. However, due to the inherent differences in the color, light, material, texture, and the realistic operating environment between the two domains, the style of the rendering image $I^{ct}$ is still quite different from the endoscopic image $I^{endo}$.

To alleviate the effect of domain shift between multimodal images, we trained a translation network to invert the style from the $I^{endo}$ to the $I^{ct}$. The cycle‐consistent generative adversarial network (CycleGAN)^26^ is adopted due to the lack of paired data sets. The CycleGAN network includes two generator networks ($G^{s\to t}$, $G^{t\to s}$) and two discriminator networks ($D^{s}$, $D^{t}$). The generator is aimed at producing fake $I^{endo}$ and $I^{ct}$ that are accurate enough to fool the discriminator. Discriminator networks are used to distinguish the generated images from “real” and then update the generator networks accordingly. This adversarial behavior is formalized through the loss function as follows:

(2) $$\begin{array}{ccc} L_{cyc}\;\left(G^{s\to t},G^{t\to s},I^{endo},I^{ct}\right) & = & E\left[{∥G^{t\to s}\left(G^{s\to t}\left(I^{endo}\right)\right)-I^{endo}∥}_{1}\right]\\ & & +\;E\left[{∥G^{s\to t}\left(G^{t\to s}\left(I^{ct}\right)\right)-\;I^{ct}∥}_{1}\right] \end{array}$$

(3) $$\begin{array}{ccc} & & L_{gan}\;\left(G^{s\to t},D^{t},I^{endo},I^{ct}\;\right)=E\left[\log D^{t}\left(I^{ct}\right)\right]\\ & & \;+\;E\left[\log\left(1-D^{t}\left(G^{s\to t}\left(I^{endo}\right)\right)\right)\right] \end{array}$$

(4) $$\begin{array}{ccc} & & L_{GAN}\;\left(G^{s\to t},G^{t\to s},D^{t},D^{s},I^{endo},I^{ct}\;\right)\\ & & \;=\;L_{gan}\left(G^{s\to t},D^{t},I^{endo},I^{ct}\;\right)\\ & & \;+\;L_{gan}\left(G^{t\to s},D^{s},I^{ct},I^{endo}\;\right) \end{array}$$

(5) $$\begin{array}{ccc} L_{base} & = & \;L_{cyc}\left(G^{s\to t},G^{t\to s},I^{endo},I^{ct}\right)\\ & & +L_{GAN}\left(G^{s\to t},G^{t\to s},D^{t},D^{s},I^{endo},I^{ct}\;\right) \end{array}$$

**FIGURE 6:**
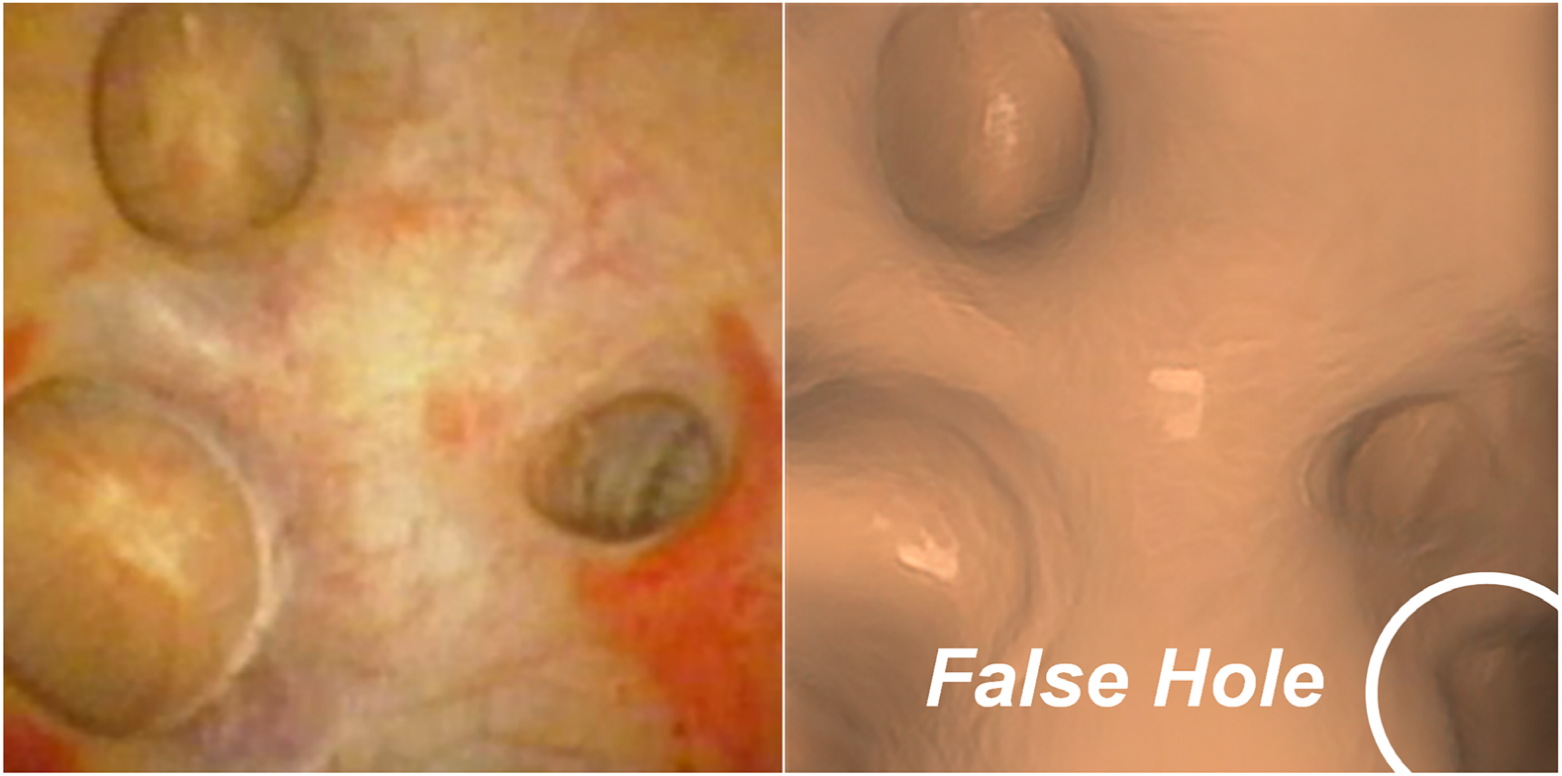
False hole generated from the cycle‐consistent generative adversarial network (CycleGAN).

However, CycleGAN has changed the intensity and anatomical shape simultaneously, so that the semantic mask of the generated image no longer corresponds to that of the original image.^27^ In our application, the network produces false holes due to the image over‐rendering, as shown in Figure 6. We introduced a semantic consistency loss $L_{sem}$ into CycleGAN to avoid geometric changes between the real and synthesized images. A semantic mask categorized into holes and wall was obtained by using a trained U‐Net.^28^ Then, a K‐way classification with a cross‐entropy loss is used to establish the semantic consistency loss as follows:

(6) $$\begin{array}{ccc} & & L_{task}\;\left(u_{s},G^{s\to t},I^{endo}\right)\\ & & =\;-E\sum_{k\;=\;1}^{K}1_{\left[k\;=u_{s}\;\left(I^{endo}\right)\right]}\log\left(\sigma \left(u_{s}^{\left(k\right)}\left(G^{s\to t}\left(I^{endo}\right)\right)\right)\right) \end{array}$$

(7) $$\begin{array}{ccc} & & L_{sem}\;\left(G^{s\to t},I^{endo},G^{t\to s},I^{ct},u_{s}\right)=L_{task}\;\left(u_{s},G^{s\to t},I^{endo}\right)\\ & & \;+\;L_{task}\left(u_{s},G^{t\to s},I^{ct}\right) \end{array}$$

**FIGURE 7:**
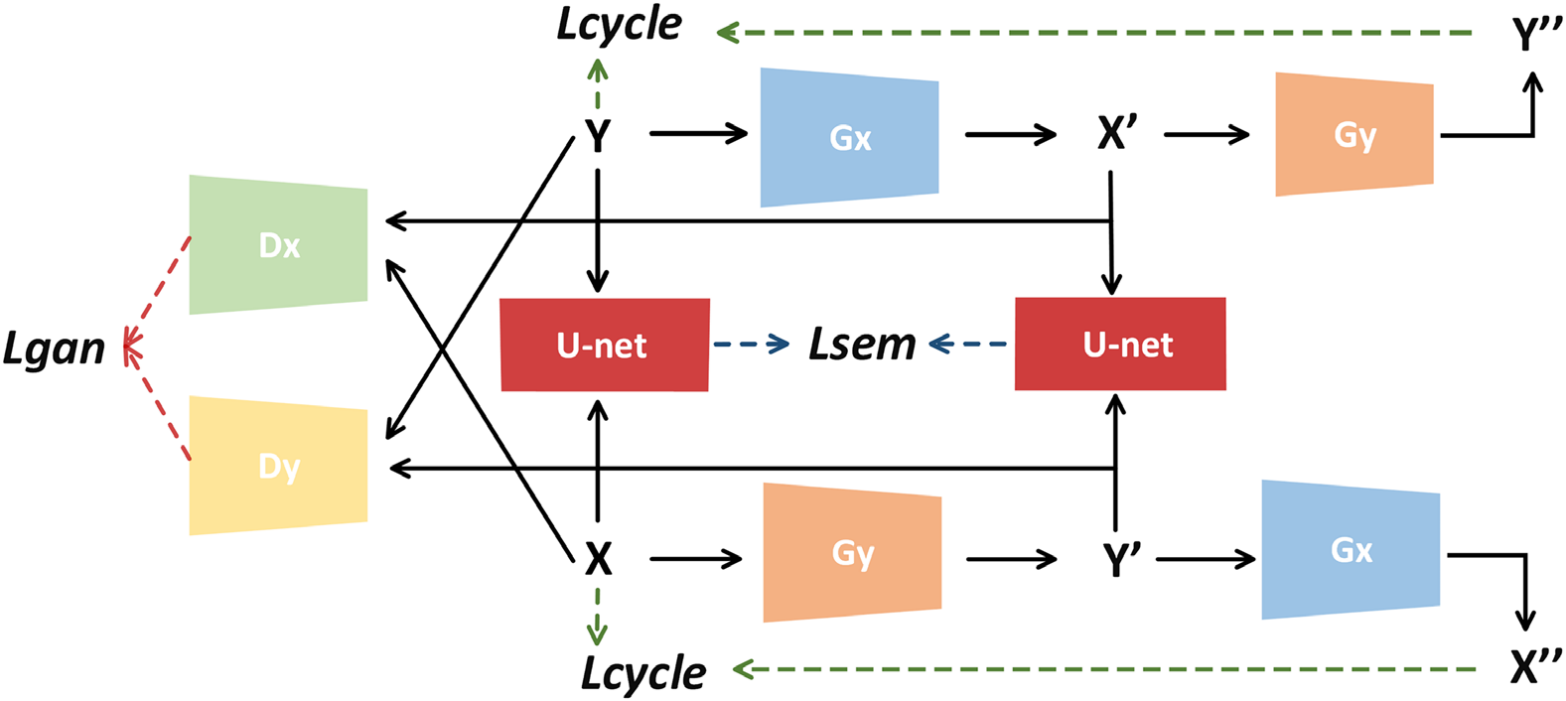
A CycleGAN‐based image translation network with semantic loss.

Taken together, the image translation network is shown in Figure 7 and its full loss functions can be summarized as

(8) $$L_{all}\left(G^{s\to t},G^{t\to s},D^{t},D^{s}\right)\;=L_{base}\;+L_{sem}$$

This ultimately corresponds to solving for a target network according to the optimization problem, which expressed as:

(9) $$t_{T}^{*}=\min_{G}\max_{D}L_{all}\left(G,D\right)\;$$

### 2.6 F.Actual contours extraction

The extraction of the actual contour of a 2D image is an important step in 2D/3D alignment. The region at contours tends to has contrasting intensity differences compared to the surroundings, which is utilized to extract contrasting regions and actual contours. It's worth noting that the extraction is performed on the 2D image $I^{ct^{\prime }}$ which is generated from the trained network $G^{s\to t}\left(I^{endo}\right)$. To improve the robustness of detection, we employ a segmentation approach SLIC^29^ as a preprocessing step to group pixels with similar characteristics to reduce the influence of abnormal single pixels.

Given a superpixel $sp_{0}$, we decide whether $sp_{0}$ is a contrasting region. It mainly includes three steps: (1) count the number of pixels $n_{sp0}$ in the $sp_{0}$ and calculate the average intensity. (2) search the neighborhood superpixel $sp_{neb}$ of $sp_{0}$ and calculate the average intensity difference, $l_{diff}=\;l_{sp0}-\;l_{sp\_neb}$. (3) obtain the maximum mean intensity difference $maxl_{0}$ and compare the $maxl_{0}$ with the threshold $\tau_{gc}$. If $maxl_{0}$ satisfies $maxl_{0}\geq \;\tau_{gc}$ and $n_{sp0}\geq \;\tau_{n}$, superpixel $sp_{0}$ is selected. Subsequently, we calculate the barycentric coordinates of selected superpixel $sp_{0}$ and record coordinate $p_{0}^{2d}\left(X,Y\right)$ as the actual contour. It is worth noting that the extracted $p_{0}^{2d}$ is likely to deviate from the actual contour coordinate in visual, but this deviation is acceptable only for supporting the subsequent fine searching.

### 2.7 G. Coarse‐to‐fine estimation

The optimal registration solution $T_{opt}$ can be represented by the optimal alignment between generated contours and the actual contours. A cost function based on similarity measure was built to guide the search for $T_{opt}$. We adopt a coarse‐to‐fine search strategy and use a Differential Evolution (DE)^30^ algorithm to search the $T_{opt}$. This process is divided into two stages: (1) iterative search of the location‐based cost function to reduce the search range; (2) under the reduced search space, optimize the cost function that contains position, gradient, and multi‐view constraints.

MI is an information‐theoretic function that measures the mutual dependence between two random variables. In the registration field, it characterizes the statistical distributions within the entire image area and is robust to noise, which is well suited for the multimodality registration scenario. In the coarse searching process, we evaluate the geometric location similarity between contours, which means attempting to align a point set $\alpha \{p_{1}\left(x,y\right),p_{2}\left(x,y\right),\text{…},p_{n}\left(x,y\right)\}$ with the other $G\{s_{1}\left(x,y\right),s_{2}\left(x,y\right),\text{…},s_{m}\left(x,y\right)\}$. The (x,y) is coordinate in 2D image. Then, a searching function of the joint probability^31^ is established by

(10) $$C_{p}\;\left(p,T\right)=\;\sum_{i\;=\;1}^{n}\sum_{j\;=\;1}^{m}p_{ij}\log\frac{p_{ij}}{\sum_{k\;=\;1}^{n}p_{kj}\sum_{l\;=\;1}^{m}p_{il}}$$

(11) $$p_{ij}=\frac{e^{-d_{ij}}}{\sum_{i=1}^{n}\sum_{j=1}^{m}e^{-d_{ij}}}\;$$

*T* represents the iterated transformation matrix, $d_{ij}$ is the Euclidean distance between $p_{i}$ and $s_{j}$. Usually, the extracted contours are at the edge with strong gradient. In the follow‐up search process, the local gradient term was added to the cost $C_{p}\left(p,T\right)$ to achieve a higher precision contour matching. The gradient term includes the gradient magnitude and orientation. To reduce the ambiguity in 2D‐2D contour matching, an additional multiple views constraint $C_{p}\left(p,T_{n}\right)$ was also integrated into the framework. $C_{p}\left(p,T_{n}\right)$ represents the contour location similarity cost calculated from the other camera pose $T_{n}$. Finally, the cost function in fine process is established by

(12) $$g_{ij}=\frac{e^{-\left(m_{ij}+o_{ij}\right)}}{\sum_{i=1}^{n}\sum_{j=1}^{m}e^{-\left(m_{ij}+o_{ij}\right)}}\;$$

(13) $$C_{grad}\;\left(g,T\right)=\sum_{i=1}^{n}\sum_{j=1}^{m}g_{ij}\log\frac{g_{ij}}{\sum_{k=1}^{n}g_{kj}\sum_{l=1}^{m}g_{il}}\;$$

(14) $$\begin{array}{ccc} C_{fine}\left(p,g,T_{0},T_{n}\right) & = & w_{1}\;*C_{p}\left(p,T_{0}\right)+w_{2}*C_{grad}\left(g,T_{0}\right)\\ & & +w_{3}*C_{p}\left(p,T_{n}\right) \end{array}$$

(15) $$T_{n}=T_{0}^{n}\;*T_{0}^{-1}$$

$m_{ij}$ and $o_{ij}$ is respectively the gradient magnitude and angle difference between *p_i_* and *s_j_*,$\;w_{1}$,$\;w_{2}$,$\;w_{3}$ represents the weight of each cost function, $T_{0}^{n}$ is the transformation between frames.

## 3 EXPERIMENTS AND EVALUATION

**FIGURE 8:**
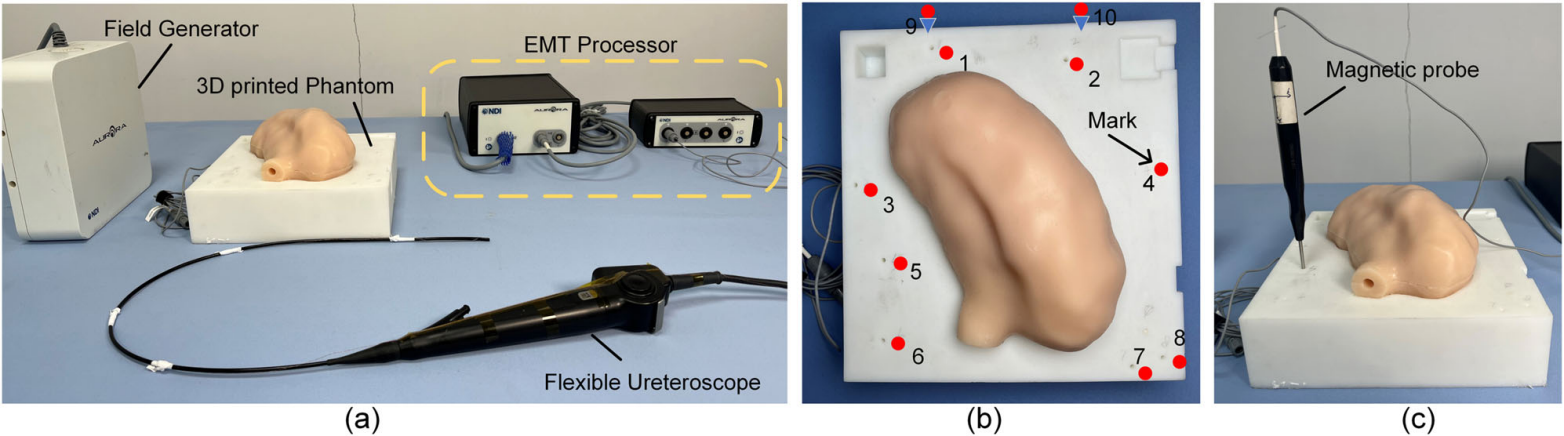
Experimental platform. (a) Data generation and collection platform. (b) A 3D‐printed phantom with ten marks. (c) The calibration between electromagnetic global and CT model coordinate system.

To validate the effectiveness of the proposed framework, we have carried out experimental verification on two phantoms based on patient CT data. We built a data acquisition platform shown in Figure 8(a, b) and used a PC (Intel®CoreTM i7‐6799K) equipped with RTX2080 (GPU, NVIDIA) to train the network and run the algorithm. Endoscopic videos were recorded using a Hawk electronic ureteroscope (DPG II) and an image processing system Hawk SD300A. An Aurora electromagnetic tracking system (NDI, Canada) and a 6 DOF electromagnetic sensor mounted on the tip of the ureteroscope were used to provide location data. The transformation between the electromagnetic tracking coordinate system and the CT volume coordinate system was calibrated, as shown in Figure 8(c).

### 3.1 Phantom and training datasets

**FIGURE 9:**
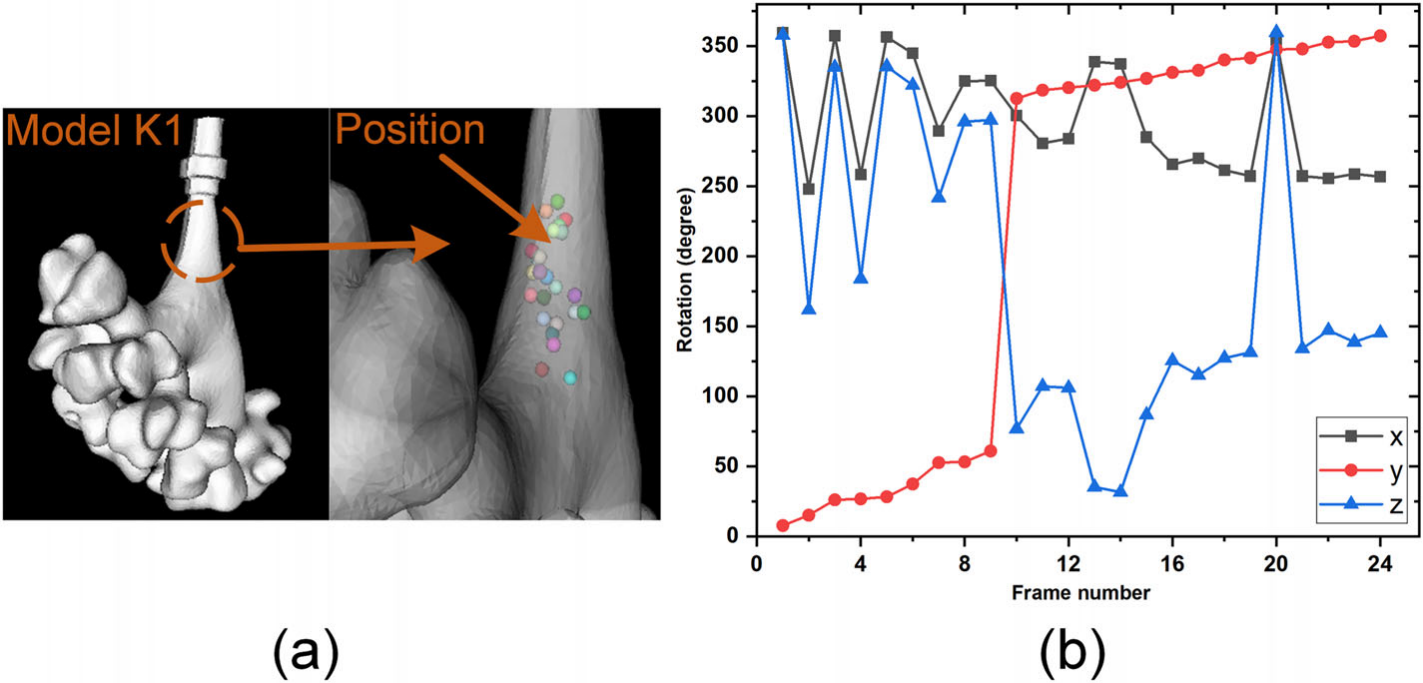
The positions and rotation of 25 sample points in K1.

**FIGURE 10:**
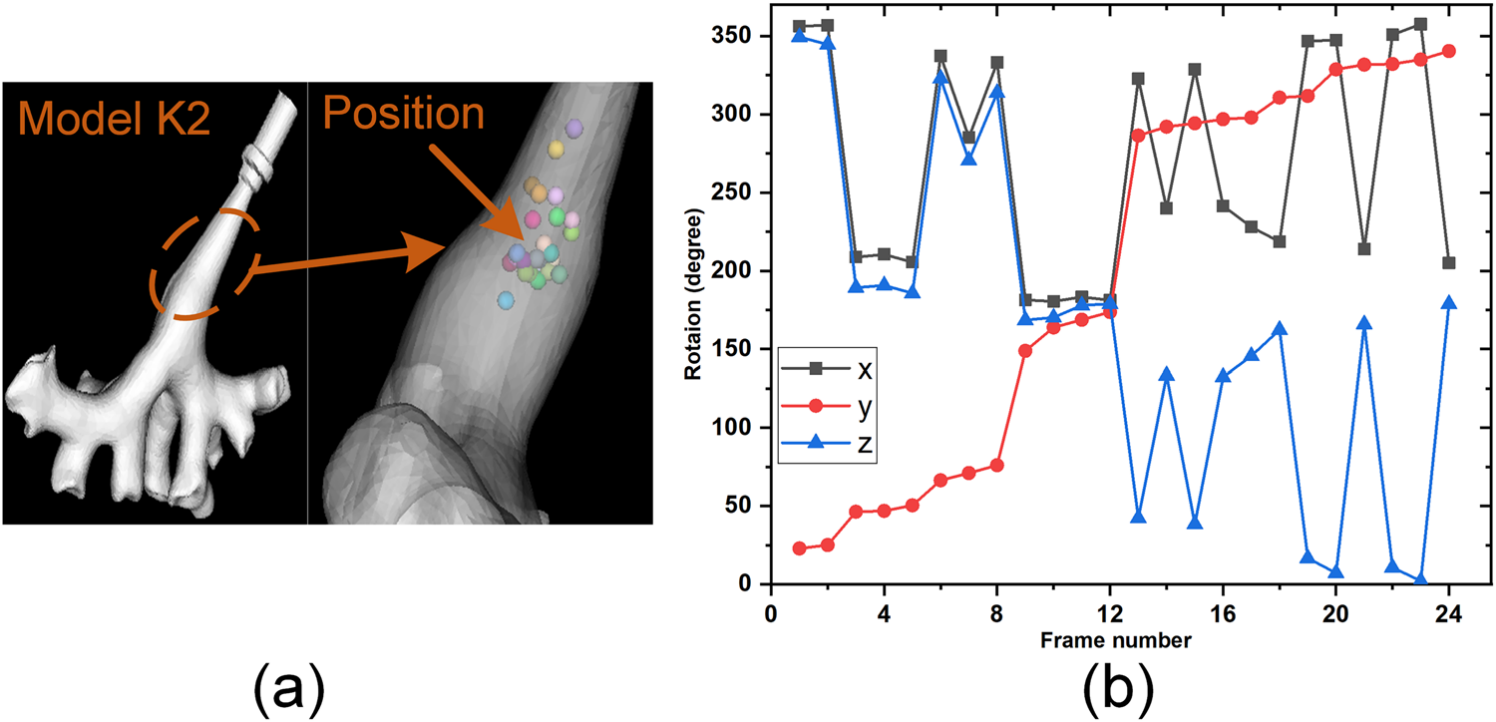
The positions and rotation of 25 sample points in K2.

Two 3D‐printed phantoms were created to collect test datasets. Each phantom contains a recessed slot for holding the model and ten markers as target points for evaluating algorithm accuracy. Marks are circular holes of 1 mm in diameter and different depth, which distribute around the kidney model, as shown in Figure 8(b). To explore the impact of structure differences on algorithm performance, the two kidney models (K1, K2) with different shapes were selected. Among them, K1 contains a complex branch structure, while the other is relatively simple, as shown in Figures 9(a) and 10(a). The CT data were acquired using a SIEMENS's device with 0.6 mm slices. To evaluate the performance of the translation network in the live conditions and the phantom conditions, we trained and tested on two datasets: live/CT and phantom/CT. The live images were collected from ten patients. The CT dataset was made by rendering from 3D printing phantoms through virtual endoscopy technology.

### 3.2 Evaluation methodology and ground‐truth

The performance of the algorithm was evaluated mainly relying on the mean target registration error (mTRE)^32^ that is defined as the distance of target points under the ground‐truth (GT) $T_{gt}$ and the estimated transformation $T_{esti}$. The mTRE is calculated by,

(16) $$\begin{array}{ccc} mTRE\left(T_{gt},\;T_{esti}\right) & = & \frac{1}{n}\sum_{i\;=\;1}^{n}Euclidean\;distance\\ & & \left(T_{gt}*p_{i},\;T_{esti}*p_{i}\right) \end{array}$$

$p_{i}$ represents the 3D position of the *i‐th* marker in the CT coordinate system. The GT $T_{gt}$ was obtained by manually adjusting the transformation matrix so that the projected contours were visual aligned with the contours on the real 2D image. Given the ambiguity of 2D matching caused by depth information collapse, it is hard to resolve the subtle deviations only relying on a visual way. We added a selection criterion $d_{em}=\;mTRE\left(T_{gt}^{em},\;T_{gt}\right)$ to filter out the optimal truth value. Among them, $T_{gt}^{em}$ represents the transformation calculated utilizing the data of electromagnetic positioning as follows:

(17) $$T_{gt}^{em}\;=T_{cami}^{emi}\;*T_{emi}^{em0}*T_{em0}^{ct}$$

$T_{em0}^{ct}$ represents the calibrated transformation from CT to NDI coordinate system, $T_{emi}^{em0}$ represents magnetic positioning information under the current frame, $T_{cami}^{emi}$ represents the rigid‐body transformation between camera and magnetic sensor coordinate system. Considering the calibration error, we set the threshold $d_{em}$ as 2.5 mm, and the smaller the value is the better if there is no distinguishable contour misalignment.

### 3.3 Search range and sampling pose

With reference to the optimization problem, we set up a 6 DOF searching range based on a start pose $\mu_{0}\left[r_{0}^{x},r_{0}^{y},r_{0}^{z},t_{0}^{x},t_{0}^{y},t_{0}^{z}\right]$. μ_0_ was calculated by manual matching 4−5 anatomical marks on the CT model and the phantom. We set an angle search range of $\left[r_{0}^{x}\pm 15^{\circ },r_{0}^{y}\pm 15^{\circ },r_{0}^{z}\pm 15^{\circ }\right]$. The search range of position was set as $\left[t_{0}^{x}\pm 10\;\mathrm{mm},t_{0}^{y}\pm 10\;\mathrm{mm},t_{0}^{z}\pm 10\;\mathrm{mm}\right]$ referring to the size of the renal pelvis. Evaluation using randomly sampled start positions is part of the standardized evaluation methodology for 2D/3D registration. In our randomized study, sampling points varied mainly in depth and roll rotation considering the surgical scene and the shape from the ureter to the renal pelvis. We collected 25 test positions in phantom K1 and K2 respectively, and their positions and orientations are shown in Figures 9 and 10.

### 3.4 Experiment results

**FIGURE 11:**
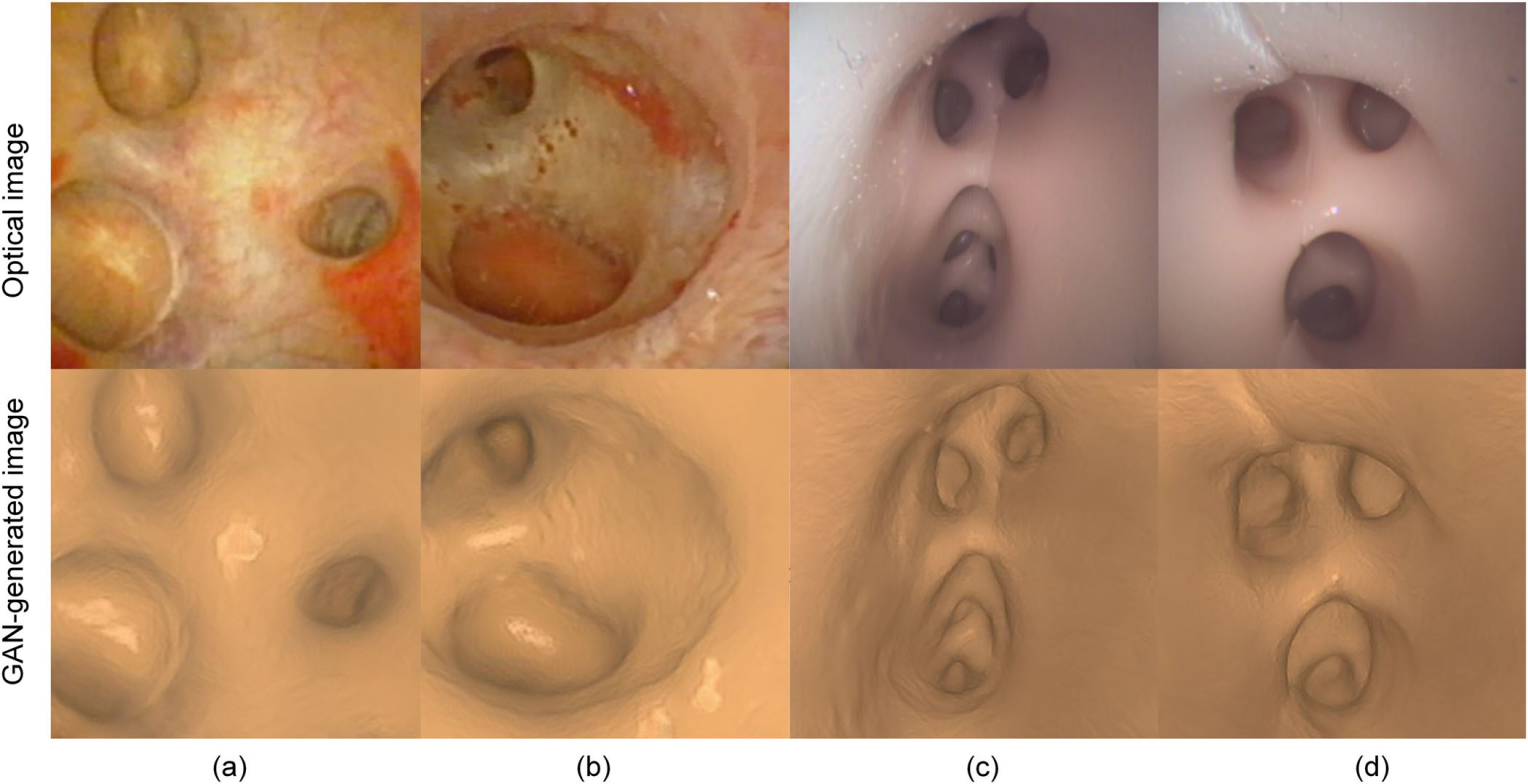
The results of the translation network. Images in (a)and(b) were from two patients. Images in (c)and(d) were from K1 and K2 models.

**FIGURE 12:**
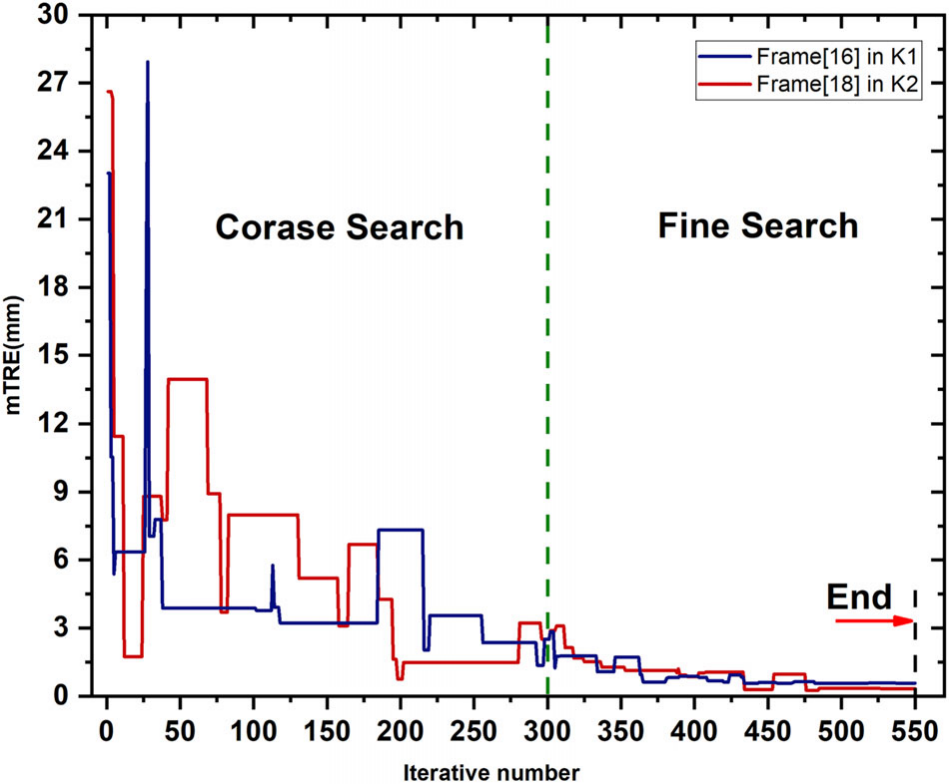
The trend of mean target registration error (mTRE) during coarse‐to‐fine iterations.

1. The semantic‐based translation network was tested on real endoscopic images and images captured from models. Some results are shown in Figure 11. The results indicate that the semantic constraints effectively inhibits the generation of false holes. To evaluate the quality of the synthetic image, the indicators of Mean absolute error (MAE), Peak‐Signal‐Noise‐Ratio (PSNR), Structural Similarity (SSIM), Multiple scales structural similarity (MS‐SSIM), and Sharpness Difference (SD) were adopted.^33^ Measurements are computed as shown in Table 1.
2. The quality of contour extraction in 2D image will directly affect the registration accuracy of the algorithm. Therefore, according to the study,^34^ a distance‐based indicator $KPI_{\Psi }\in \left[0,1\right]$ was selected as the measure to evaluate the accuracy and robustness of our auto‐contouring method. The value $KPI_{\Psi }$ close to 0 indicates a good segmentation, otherwise, the value close to 1 for a poor segmentation. The contour GT was obtained by manual labeling. For phantom experiments, we compared the difference between projected contours calculated from GT registration matrix and extracted contours from cycleGAN 2D images. For contour quality evaluation in real images, we compared the difference of results with and without the cycleGAN. The results are shown in Table 2. The results show that the contour extraction method in this paper achieves acceptable $KPI_{\Psi }$ values (all less than 0.5) and performs well on phantoms. The contour extraction error of the real image is higher than that of the phantom, but it is still below 0.5, and the extraction results using the cycleGAN network is better than that directly from the original image.
3. We evaluated the performance of the proposed method and compared it with five competing methods used in 2D/3D registration for endoscope: NMI,^20^ $MI_{grad}$,^23^ RANSAC‐PnP,^19^ MS‐DSSM^25^ and CycleGAN‐Depth.^15^ The Powell algorithm was used to optimize the competing method, and the initial value of the optimization was set to μ_0_. We calculated the mean and standard deviation on indicators such as mTRE, orientation error (OE), and position error (PE). The initial registration errors mTRE obtained on K1 and K2 phantom are respectively 3.79±0.78mm and 7.61±0.63mm.The comparison results with other methods are shown in Table 3 and Table 4. In K1 cases, the mean and standard deviation of the mTRE, OE, and PE are respectively0.97±0.59 mm, $0.54^{\circ }\pm 0.20^{\circ }$and 0.42 ± 0.24 mm. In K2 cases, the mean and standard deviation of the mTRE, OE, and PE are respectively 1.27 ± 0.42 mm, $0.73^{\circ }\pm 0.25^{\circ }$and 0.58 ± 0.21 mm. The results indicate that the proposed method significantly improved the accuracy and robustness. In Figure 12, we show the trend of mTRE during coarse‐to‐fine iterations, which is in line with our expectations. After reducing the search range through iterations in the coarse registration stage, higher precision registration is achieved through introducing local gradients.
4. Ablation study: the cycleGAN and contour matching are two important modules in our algorithm. Therefore, the ablation experiments were performed in two groups to verify the effectiveness: without the cycleGAN and without the contour matching. The comparative experimental results are shown in Table 5. The results show that contour matching contributes more to the accuracy of the algorithm, but style transfer network can also improve the performance.

**TABLE 1:** Quantitative evaluations on image style transfer network.

Category	MAE	PSNR	SSIM	MS‐SSIM	SD
K1	24.16 ± 4.32	19.18 ± 1.41	0.91 ± 0.01	0.69 ± 0.05	32.04 ± 0.28
K2	19.25 ± 4.73	21.18 ± 1.84	0.92 ± 0.02	0.69 ± 0.07	32.17 ± 0.37
real	34.60 ± 13.25	16.99 ± 3.10	0.84 ± 0.04	0.70 ± 0.08	30.42 ± 0.64

**TABLE 2:** Quantitative evaluations on contour quality.

	**K1**		**K2**		**real**	
**Indicator**	detect	project	detect	project	original	cycleGAN
KPI_Ψ_	0.12 ± 0.04	0.02 ± 0.02	0.09 ± 0.03	0.02 ± 0.02	0.37 ± 0.22	0.27 ± 0.18

**TABLE 3:** The registration results of the comparison experiments on the K1 model.

	K1					
Indicators	Our	NMI	${\mathrm{MI}}_{grad}$	PnP	MS‐DSSIM	Depth‐GAN
mTRE (mm)	**0.97 ± 0.59**	8.48 ± 4.15	4.70 ± 1.57	52.18 ± 25.65	13.79 ± 6.96	11.82 ± 6.08
OE (°)	**0.54 ± 0.19**	5.35 ± 1.82	3.07 ± 1.09	37.96 ± 33.92	8.64 ± 4.16	6.86 ± 3.37
PE (mm)	**0.42 ± 0.24**	2.63 ± 0.74	2.11 ± 0.72	17.28 ± 12.07	3.95 ± 2.36	4.90 ± 2.33

**TABLE 4:** The registration results of the comparison experiments on the K2 model.

	K2					
Indicators	Our	NMI	${\mathrm{MI}}_{grad}$	PnP	MS‐DSSIM	Depth‐GAN
mTRE (mm)	**1.27 ± 0.42**	16.24 ± 14.12	8.69 ± 3.59	81.14 ± 72.65	14.56 ± 6.43	14.43 ± 5.56
OE (°)	**0.54 ± 0.19**	5.35 ± 1.82	6.14 ± 2.34	46.82 ± 48.40	9.27 ± 3.70	8.20 ± 2.94
PE (mm)	**0.42 ± 0.24**	2.63 ± 0.74	3.93 ± 2.23	41.28 ± 50.92	4.58 ± 2.44	5.12 ± 1.92

**TABLE 5:** Ablation study.

	K1			K2		
Indicators	A+B+C+D	A+C	B+C+D	A+B+C+D	A+C	B+C+D
mTRE (mm)	**0.97 ± 0.59**	27.14 ± 23.46	3.49 ± 5.85	**1.27 ± 0.42**	17.62 ± 6.17	2.38 ± 1.11
OE (°)	**0.54 ± 0.19**	15.99 ± 15.18	2.08 ± 3.65	**0.73 ± 0.25**	10.76 ± 4.22	1.46 ± 0.66
PE (mm)	**0.42 ± 0.24**	6.99 ± 3.40	1.31 ± 1.84	**0.58 ± 0.21**	3.70 ± 1.64	0.74 ± 0.44

## 4 DISCUSSION AND CONCLUSION

We developed and investigated a 2D/3D registration method used in RIRS. Structures of interest are extracted and projected into the generated contours to match with the actual contours. A translation network and multi‐view constraint were integrated to achieve higher precision registration. The performance of the method was evaluated in phantoms and compared with other endoscopic registration techniques. The results show that our approach has a better accuracy and variability overall. The indicators on model K1 are better than that of model K2. One possible reason is that the more complex lumen structure of K1 brings more structural constraints.

**FIGURE 13:**
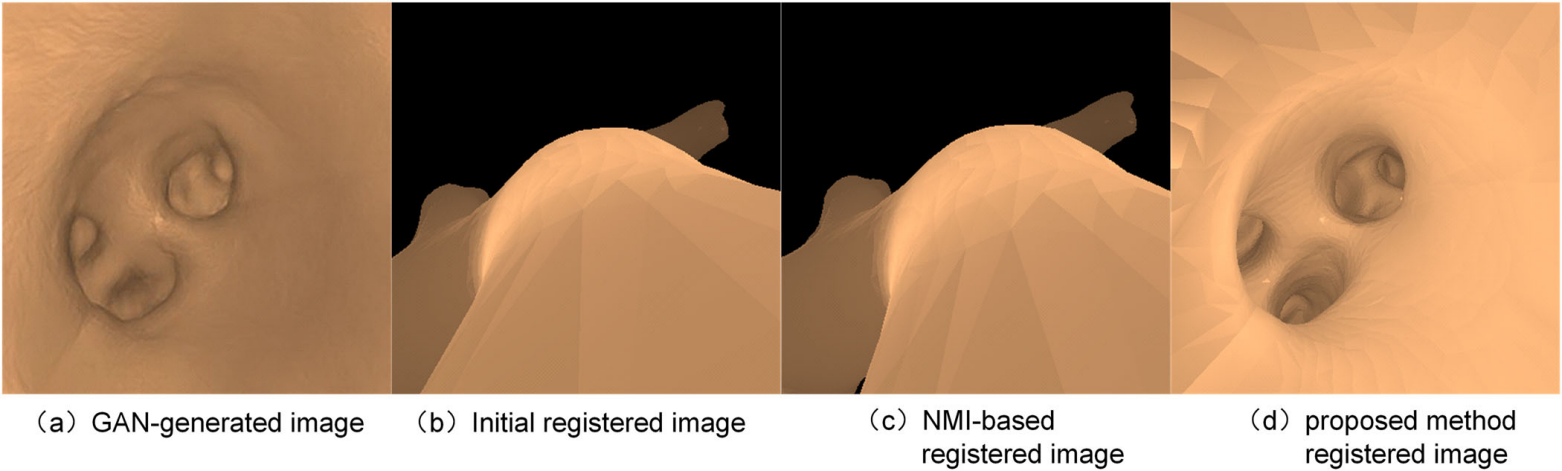
Comparison of results when initial positions outside of the model. (a) is the GAN‐generated image translated from the real image.

Intensity‐based registration is usually greatly affected by the intensity differences between images. A translation network was trained and used to alleviate the impact of intensity differences. The results show that the translation network improved indicators. Due to the lack of structural constraints and the influence of initial values, the competing algorithms tend to have considerable bias and randomness. As shown in Figure 13, when the initial position is outside of the model, the optimized outcomes are still outside. The introduced maximum contour position similarity constrains the solution space, so that our algorithm can run in a rough search space, which suppresses the generation of the above‐mentioned bad results.

Although the proposed method performs well on phantoms, it still has some limitations. The quality of extraction of actual contours has a significant impact on registration accuracy. Fortunately, the translation network can be trained to generate images that salient regions can be extracted more easily. Another disadvantage is that the running time is about 30 min. The time cost mainly comes from the image processing in the fine search stage, such as gradient calculation and rendering. Parallel computing can be considered to optimize algorithm and reduce the computation time. This is also the focus of our follow‐up research work. Finally, we found that binary classification semantic loss suppresses the appearance of false holes, but it can't solve them completely. One of the solutions may be multi‐label classification and image recognition. Moreover, the initial TRE on phantom results would better than the actual patient's TRE. Therefore, this may have two effects on the actual registration process when setting up range centering on *T*_0_ : 1) When the search space is too small and the true value is not included in it, the algorithm may generate a poor result; 2) When the search space is expanded, a local optimum may appear. Therefore, in order to verify the effectiveness, we expanded the search range to test the performance of the algorithm. The comparison results are shown in Table 6, in which ‘[15,10]’ denotes ±$\;15^{\circ }$ in rotation, ± 10 mm in translation, ‘[90,30]’ denotes ±$\;90^{\circ }$ in rotation, ± 30 mm in translation. We found that our algorithm still maintains a good registration accuracy compared with small search range (mTRE is increased within 1 mm). However, the average number of iterations required increased (from 550 to 950) when the solution range increased. Therefore, more in vivo experiments are needed to verify the accuracy and robustness of the method.

**TABLE 6:** The results of the comparison experiments on an expanded search range.

	K1		K2	
Indicators	[15,10]	[90,30]	[15,10]	[90,30]
mTRE (mm)	0.97 ± 0.59	1.62 ± 0.97	1.27 ± 0.42	1.97 ± 0.85
OE (°)	0.54 ± 0.19	0.96 ± 0.61	0.73 ± 0.25	1.67 ± 2.62
PE (mm)	0.42 ± 0.24	0.66 ± 0.39	0.58 ± 0.21	1.19 ± 2.01

Overall, the proposed method has the potential to support registration in RIRS and be extended to other organs with similar structures. To further enhance the robustness and effectiveness, the quality of contour generator needs to be improved to cope with more complex intraoperative environments. Required work includes collecting more realistic surgical images, classifying image content more precisely, and training style transfer networks to extract contours more efficiently.

## AUTHOR CONTRIBUTIONS

Zuoming Fu: Drafting the manuscript, Methodology, Software, Validation. Ziyi Jin: Methodology, Review & Editing. Chongan Zhang: Methodology, Review & Editing, Validation, Investigation. Peng Wang: Supervision, Data Curation, Acquisition of data, Review & Editing. Hong Zhang: Funding acquisition, Review & Editing. Xuesong Ye: Review & Editing, Funding acquisition, Project administration, Revising the manuscript critically for important intellectual content.

## CONFLICT OF INTEREST STATEMENT

The authors declare that they have no conflict of interest.

## ETHICAL APPROVAL

This article does not contain any studies with human participants or animals performed by any of the authors.
